# Long-term improvement of quality of life in patients with breast cancer: supporting patient-physician communication by an electronic tool for inpatient and outpatient care

**DOI:** 10.1007/s00520-021-06270-1

**Published:** 2021-06-27

**Authors:** Patricia Lindberg-Scharf, Brunhilde Steinger, Michael Koller, Andrea Hofstädter, Olaf Ortmann, Jan Kurz, Jonathan Sasse, Monika Klinkhammer-Schalke

**Affiliations:** 1 Tumor Center Regensburg, Institute of Quality Management and Health Services Research of the University of Regensburg, Am Biopark 9, 93053 Regensburg, Germany; 2 Center for Clinical Studies, University Hospital Regensburg, Franz-Josef-Strauß-Allee 11, 93053 Regensburg, Germany; 3 Department of Obstetrics and Gynecology, University Hospital Regensburg, St. Hedwig Clinic, Steinmetzstraße 1-3, 93049 Regensburg, Germany; 4 Department of Gynecology and Obstetrics, University Medical Center Regensburg, Landshuter Straße 65, 93053 RegensburgRegensburg, Germany

**Keywords:** Quality of life, Patient-reported outcomes, Breast cancer, Electronic assessment, Patient-physician communication, Complex intervention

## Abstract

**Purpose:**

The effectiveness of a pathway with quality of life (QoL) diagnosis and therapy has been already demonstrated in an earlier randomized trial (RCT) in patients with breast cancer. We refined the pathway by developing and evaluating an electronic tool for QoL assessment in routine inpatient and outpatient care.

**Methods:**

In a single-arm study, patients with breast cancer with surgical treatment in two German hospitals were enrolled. QoL (EORTC QLQ-C30, QLQ-BR23) was measured with an electronic tool after surgery and during aftercare in outpatient medical practices (3, 6, 9, 12, 18, and 24 months) so that results (QoL-profile) were available immediately. Feedback by patients and physicians was analyzed to evaluate feasibility and impact on patient-physician communication.

**Results:**

Between May 2016 and July 2018, 56 patients were enrolled. Physicians evaluated the QoL pathway as feasible. Patients whose physician regularly discussed QoL-profiles with them reported significantly more often that their specific needs were cared for (*p* < .001) and that their physician had found the right treatment strategy for these needs (*p* < .001) compared with patients whose doctor never/rarely discussed QoL-profiles. The latter significantly more often had no benefit from QoL assessments (*p* < .001).

**Conclusion:**

The QoL pathway with electronic QoL assessments is feasible for inpatient and outpatient care. QoL results should be discussed directly with the patient.

**Clinical trial information:**

NCT04334096, date of registration 06.04.2020

**Supplementary Information:**

The online version contains supplementary material available at 10.1007/s00520-021-06270-1.

## Introduction

Patients with cancer considerably suffer from impairments of their quality of life (QoL) during diagnosis, therapy, and aftercare. Hospitals more and more focus on additional supportive care (e.g. psycho-oncological care, nutritional therapy). However, patients usually stay in the hospital only for a few days whereas many QoL impairments appear in the later course of the disease during adjuvant therapy and aftercare [1, 2].

Studies have shown that structured QoL interventions for routine care are well accepted by patients and physicians [3–5], and improve patient-physician communication [6–8], QoL [7, 9], and even survival [9, 10]. A major limitation of these studies is that interventions were limited to the inpatient setting or to a single institution for outpatient care so that there is a lack of generalizability of these findings. Therefore, the Tumor Center Regensburg developed a QoL pathway with systematic QoL diagnosis and tailored QoL therapy for inpatient and outpatient care in a complex intervention [11, 12]. Two randomized trials (RCTs) in patients with breast [13] and colorectal cancer [14] demonstrated effectiveness by showing a significantly better QoL in the intervention group patients. In both trials, QoL was measured with a paper–pencil questionnaire that was entered by hand into an electronic database to obtain results. Therefore, patients and physicians could discuss results only with a delay of about 1 week after measurement.

Electronic tools for the automatic assessment of patient-reported outcomes have been developed, implemented, and evaluated in numerous studies [7–10]. Above all, such interventions have the advantage that results are available in real time for patients and physicians and can be directly discussed. However, there is a lack of feasibility for routine outpatient care because most medical practices do not have the necessary technical equipment for their patients such as tablet computers or computer workplaces.

Therefore, the aim of the present study was to overcome this problem by refining the QoL pathway so that QoL results could be electronically processed and presented in real time to patients and physicians in inpatient and outpatient care without the need for additional technical equipment such as tablet computers or smartphones in outpatient practices. Feasibility of the QoL pathway and its impact on patient-physician communication were analyzed.

## Methods

### Study design

The study was designed as a prospective, single-arm, clinical trial of a complex intervention including a one-group pretest–posttest design. Moreover, QoL data of the present study were compared with those of an earlier RCT [13] that investigated the effectiveness of the QoL pathway for patients with breast cancer.

### Participants and setting

Patients were enrolled by study clinicians of two participating German Cancer Society (DKG)-certified breast cancer centers in Bavaria, Germany (Department of Gynecology and Obstetrics, University Medical Center Regensburg; Department of Obstetrics and Gynecology of the University Medical Regensburg, St. Hedwig Clinic). Inclusion criteria were diagnosis of breast cancer, with surgical treatment in one of the two hospitals, and informed consent to participate in the study. Exclusion criteria include the following: (1) recruiting study clinician unavailable; (2) patient misclassified in the operation schedule (no breast neoplasm); (3) coordinating practitioner refused trial participation; (4) no “Nachsorgekalendernummer” available (unique number of the diary a patient with cancer receives in the hospital for aftercare); (5) patient from district outside the defined study region; (6) age under 18 years; (7) pregnancy; (8) patient unable to fill out QoL questionnaires (physical, psychological, cognitive, language reasons); and (9) patient refused trial participation.

Aftercare was conducted in the outpatient practices of the patients’ coordinating practitioners (CP: gynecologist responsible for aftercare). Patients were free to choose their CP. CPs of patients who agreed to participate were contacted by the recruiting clinician and asked to participate in the trial. The two study coordinators (PLS psychologist, BS gynecologist) individually trained each CP in an educational outreach visit in his/her practice by providing information about the aims and the procedure of the study. No additional study case-based payments were provided.

### Intervention

**Fig. 1:**
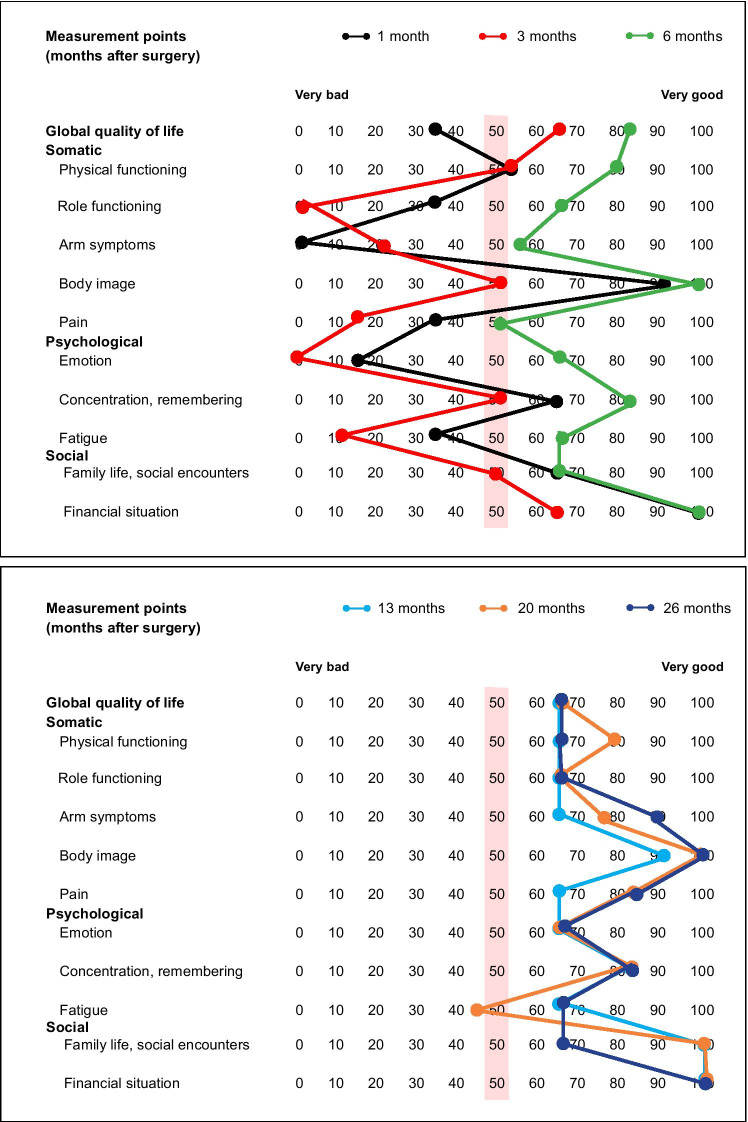
Quality of life (QoL-) profile: woman with primary breast cancer, 49 years, married, one child, working. Prognostic classification pT1b(m), SN0, M0, G1, ER pos, PR pos, HER2 neg; breast-conserving surgical therapy with revision surgery after 1 month followed by radiation and anti-estrogen treatment. Red bar = cutoff for a need for QoL therapy (< 50 points)

All patients repeatedly filled out QoL questionnaires during the first 2 years after surgery. CPs received QoL results of their patients added by an address list with local healthcare professionals for specific QoL therapies. QoL data were automatically processed with the electronic data processing (EDP)-aided system “*LPro*”:

1. *Inpatient QoL diagnosis: tablet computer*
  In the hospital, a clinician respectively study nurse conducted the first QoL measurement (baseline) when the patient came for routine consultation 1 week after discharge. Patients answered the QoL questionnaire on a tablet computer. A presentation of one question per screen was chosen to allow for better focus and the duration of time to fill out the questionnaire was automatically recorded. A final question asked for technical problems. After finishing data entry, a QoL profile (see Fig. 1) with results was displayed on the screen so that the clinician could discuss the profile immediately with the patient. Simultaneously, the profile was sent via e-mail to the authorized clinicians of the hospital and the study coordinators. Alternatively, patients could also complete a paper–pencil version of the QoL questionnaire based on preference as studies have shown that results of electronic and paper–pencil measurements are equivalent [15, 16]. However, in this case the QoL-profile was not available in real-time.
2. *Outpatient QoL diagnosis: EDP-aided paper-based assessment*

Further QoL measurements were conducted in the practice of the CP in accordance with the national practice guideline for breast cancer at 3, 6, 9, 12, 18, and 24 months [17]. Usually, outpatient practices do not have a tablet computer or computer workplace for patients. Therefore, an EDP-aided paper-based method was developed (see Supplementary Fig.): CPs received a paper–pencil version of the QoL questionnaire. An individual QR code with pseudonymized patient information was printed on each questionnaire ensuring that QoL data were assigned to the right patient. For each assessment time point, participants received a postal reminder. The questionnaire was given to the patient during her visit in the practice to be completed during waiting. After that, the questionnaire was sent to a local digital fax server where it was automatically processed and answers were stored in a database. Following this, a QoL-profile was generated and transferred back to the CP’s practice via e-mail or fax (depending on CP’s preference). The processing time took on average 17.43 s (SD = 23.17). In the Supplementary Information, a detailed description of server configuration and used software can be found. The immediate response enabled patients and CPs to discuss the QoL-profile directly. If a patient`s QoL was below 50 points in at least one dimension of the QoL profile the CP automatically received an address list with local healthcare providers of a multiprofessional care network.

#### Multiprofessional network for inpatient and outpatient care

For the tailored treatment of QoL, a regional network structure of healthcare providers for inpatient and outpatient care had already been established during the two earlier RCTs [13, 14]. This care network encompassed a total of 54 therapists providing the following different therapeutic options:

- physiotherapy (*n* = 9),
- psychotherapy (*n* = 12),
- pain therapy (*n* = 6),
- social support (*n* = 6),
- nutritional counseling (*n* = 14),
- fitness (*n* = 7)

For each therapeutic option, therapists regularly met in quality circles. All professionals were certified in their respective field (e.g., certified psycho-oncologist or certified stoma nurse). CPs received contact information of quality circle members to contact them for QoL therapy.

### Measures

#### Quality of life

Quality of life was measured with the European Organization for Research and Treatment of Cancer (EORTC) core questionnaire QLQ-C30 (version 3.0) [18] and the breast cancer-specific module EORTC QLQ-BR23 [19]. EORTC QLQ-C30 aggregates 30 items into six functioning scales, three symptom scales, and five single items. The EORTC QLQ-BR23 consists of 23 items that are aggregated into two functional scales, three symptom scales, and three single items. All scores were linearly transformed and presented on scales from 0 to 100 [20]. We used a uniform manner for transformation with 0 denoting the negative (low functioning, high symptom burden) and 100 the positive end (high functioning, low symptom burden) of the continuum [21]. The QoL profile (see Fig. 1) shows a patient’s QoL on eleven scales of the QLQ-C30 and QLQ-BR23. We had selected these scales based on the experience of the previous RCT [13], appraisal of relevance, and the availability of specific therapies to improve QoL. A cutoff score < 50 points defined a “need for QoL therapy”. This decision criterion was chosen to highlight the patient`s perspective of subjective wellbeing by dichotomizing symptom scores with a majority of “quite a bit” and “very much” responses to the “bad” side of the spectrum (< 50) and “not at all” and “a little” responses to the “good” side (≥ 50) [13, 22, 23]

#### Health status

The recruiting clinician documented the following demographic and clinical patient variables at study entry: age, marital status, employment status, number of children, tumor stage, primary disease, receptor status, date of surgery, surgical procedure, comorbidities, and neoadjuvant therapy.

#### Patient evaluation

At 6 and 24 months after surgery, patients received a paper-based self-developed questionnaire with quantitative questions asking for feedback about the usefulness of the QoL pathway from their point of view.

#### Physician evaluation

At 24 months postoperatively, CPs received a paper-based self-developed questionnaire with quantitative questions asking for feedback about the feasibility, acceptability, and usefulness of the QoL pathway.

The management and storage of data were in accordance with the European General Data Protection Regulation (GDPR). The trial was approved by the ethics committee of the University of Regensburg (reference number 15–101-0320).

### Statistical analysis

All statistical comparisons were two-tailed and a *p* value of < 0.05 indicated statistical significance. Continuous variables are presented as means (standard deviation) and categorical variables as absolute and relative frequencies. A need for QoL therapy was defined as a score < 50 points in at least one of eleven dimensions of the QoL-profile. Rates of patients with a need for QoL therapy and rates of patients with a score < 50 points in each single QoL dimension were compared for 0 and 6 months with McNemar tests. This timepoint was chosen as it has been the primary endpoint in the previous RCT [13]. Rates of patients with a need for QoL therapy in the present study were compared with rates of two historical controls of this RCT (namely the RCT`s intervention and control group) with *χ*^2^ tests. For these comparisons, the scale “financial functioning” was excluded because this scale was not part of the RCT. All analyzes were performed using SPSS version 25.0 (IBM Corp., Armonk, NY, USA).

## Results

Between 30 May 2016 and 18 July 2018, 88 patients who fulfilled inclusion criteria were invited to participate in the trial. Of those, 56 (64%) agreed and were included in the study. Table 1 shows baseline characteristics of the study sample. Patients who refused (*n* = 32) were significantly older (mean age 66.50 vs. 50.63, *p* < 0.001), and the mastectomy rate was significantly higher (36% vs. 9%, *p* = 0.002). All participants provided written informed consent.

**Table 1:** Baseline characteristics of participants at study entry

	**No. (%) of participants (*n* = 56)**
Age (years) mean (SD)	50.63 (9.68)
Marital status *n* (%)	
Married	35 (63)
Unmarried	10 (18)
Divorced	4 (7)
Separated	2 (4)
Widowed	2 (4)
Unknown	3 (5)
Children *n* (%)	
Yes	39 (70)
No	11 (20)
Unknown	6 (11)
Employment status *n* (%)	
Employed	30 (54)
Retired/not employed	26 (46)
Prognostic stage at diagnosis *n* (%)	
UICC 0	1 (2)
UICC I	21 (38)
UICC II	27 (48)
UICC III	6 (11)
UICC IV	1 (2)
Primary disease *n* (%)	
Yes	54 (96)
No	2 (4)
Surgical procedure *n* (%)	
Breast conserving therapy	51 (91)
Mastectomy	5 (9)
Estrogen receptor positive *n* (%)	45 (80)
Progesterone receptor positive *n* (%)	44 (79)
HER2 positive *n* (%)	9 (16)
Comorbidities *n* (%)	
Cardiovascular	6 (11)
Lung	5 (9)
Kidney	1 (2)
Central nervous system	2 (4)
Neoadjuvant therapy *n* (%)	
Chemotherapy	14 (25)
None	39 (70)
Unknown	3 (5)

### Questionnaire completion mode

In the hospital, 82% (46/56) of participants completed the first QoL measurement on the tablet computer whereas 14% (8/56) preferred the paper-based questionnaire (unknown 4%, 2/56). The mean time for completion of the QoL questionnaire on the tablet computer was 9.9 min (SD = 3.4 min, range 4.9–19.0 min). Of those patients who used the tablet computer, 91% (42/46) reported no technical problems, 7% (3/46) a little, and 2% (1/46) quite a bit. No participant had serious technical problems. However, 39% (18/46) of women were supported by medical staff when entering data for unknown reasons. Those patients did not differ in their mean age (51.6 years) from women who had no support for questionnaire completion (50.5 years) or who preferred the paper-based questionnaire (52.0 years) (*p* = 0.421). During aftercare 75% of QoL questionnaires were transferred by fax, 23% by post, and 2% were completed on the tablet computer in the hospital.

### Questionnaire completion rates

**Fig. 2:**
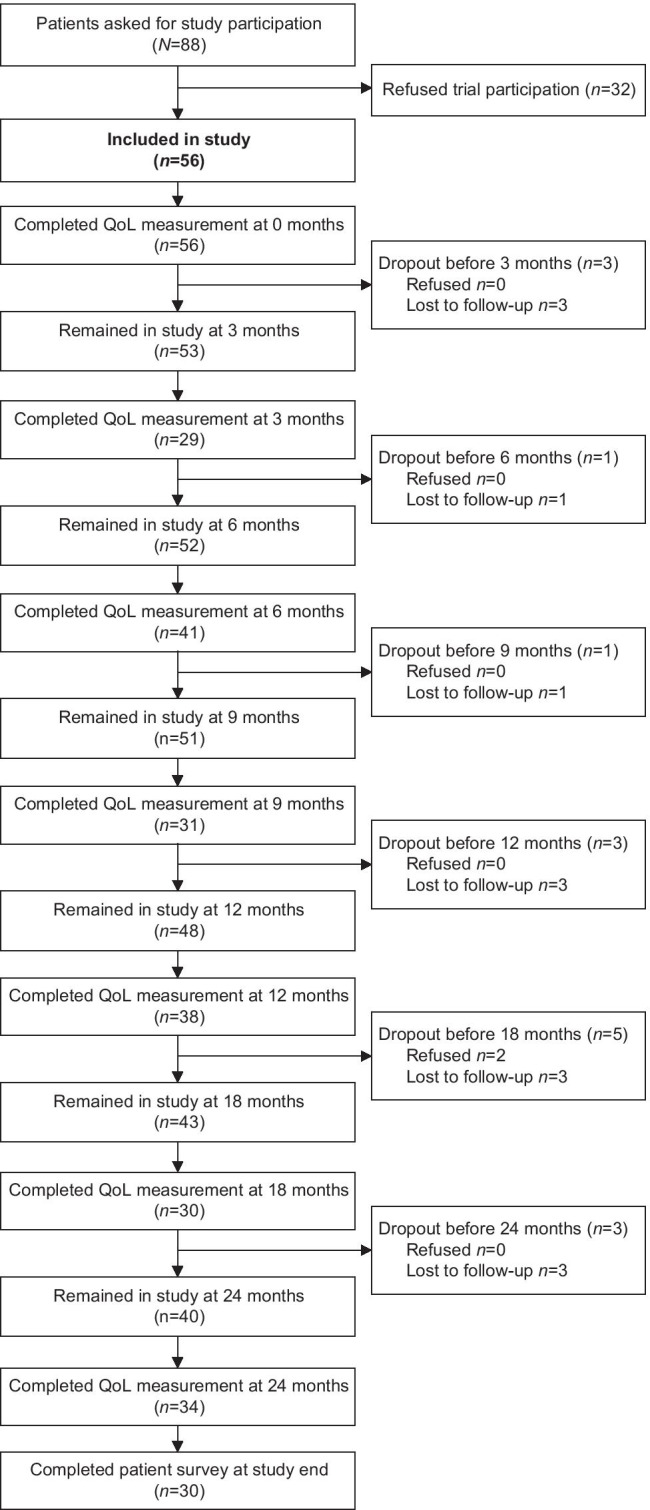
Flow chart

Figure 2 shows questionnaire completion rates during the 24-month study period. After 12 months, two women refused further trial participation because they felt well with no need for QoL measurements anymore. Seven patients were lost to follow up during the first year of the study and six during the second year. During aftercare the questionnaire completion rate was lowest at 3 months (*n* = 29) and highest at 6 months (*n* = 41). The last QoL measurement at 24 months was completed by 34 women.

### Participation of CPs

In total, 34 CPs were approached of whom 33 (97%) participated in the trial. Of those, one CP dropped out during the course of the study due to a lack of time.

### Rates of patients with a need for QoL therapy

A comparison of rates of patients with a need for QoL therapy at 0 and 6 months postoperatively revealed a non-significant reduction from 70% (28/40) to 63% (25/40) (*p* = 0.581). In single QoL dimensions, rates significantly decreased in the dimension “arm symptoms” (38% vs. 15%, *p* = 0.01) and increased in the dimension “financial impact” (8% vs. 28%, *p* = 0.01) at 6 months. On the other scales, there were no significant differences between 0 and 6 months (see Table 2).

**Table 2:** Comparison of rates of patients with a need for QoL therapy at 0 and 6 months after surgery

	**0 months**	**6 months**	***p***^**c**^
Total^a^	70%	63%	.58
Single QoL dimensions^b^			
Global QoL	29%	22%	.61
Physical functioning	7%	5%	1.00
Role functioning	34%	29%	.79
Pain	32%	24%	.58
Body image	12%	20%	.38
Arm symptoms	38%	15%	**.01**
Emotional functioning	29%	34%	.75
Cognitive functioning	12%	22%	.29
Fatigue	34%	44%	.34
Social functioning	22%	25%	1.00
Financial impact	8%	28%	**.01**

^a^Need for QoL therapy (QoL < 50 points) in at least one of eleven QoL dimensions of EORTC QLQ-C30, QLQ-BR23

^b^Need for QoL therapy (QoL < 50 points) in single QoL dimensions of EORTC QLQ-C30, QLQ-BR23

^c^All *p* values derived from McNemar tests

*p* values < 0.05 are presented in bold face

**Fig. 3:**
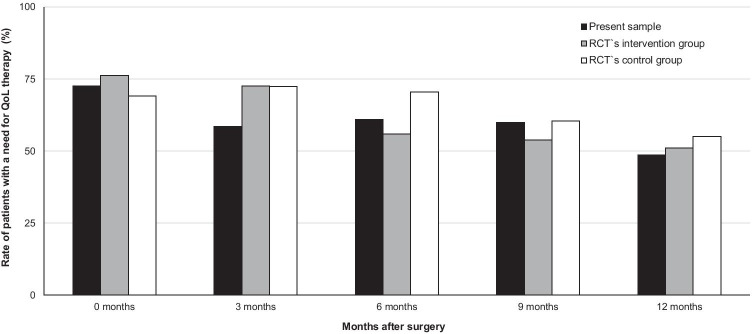
Rates of patients with a need for quality of life (QoL) therapy (QoL < 50 points on at least one of ten scales) in the present sample compared with two historical controls of the previous RCT (namely RCT’s intervention and control group) [13] over 12 months; scale “financial functioning” was excluded from the analyzes because this scale was not part of the RCT; rates at 18 and 24 months are not analyzed because these were not assessed in the RCT; *χ*^2^ tests: 0 months *p* = .54, 3 months *p* = .32, 6 months *p* = .14, 9 months *p* = .66, 12 months *p* = .77

Moreover, QoL data during the first 12 months after surgery were compared with those of the former RCT`s intervention (*n* = 99) and control group (*n* = 100) [13]. Participants of the present study (mean age 50.63 years, SD = 9.68) were significantly younger than women of both historical controls (RCT’s intervention group 58.63 years, SD = 12.09; RCT’s control group 56.75 years, SD = 11.92, *F* = 8.84, *p* < 0.001) with a higher rate of breast conserving therapy (91% vs. RCT`s intervention group 75% vs. RCT`s control group 76%; *p* = 0.039). As shown in Fig. 3, rates of patients with a need for QoL therapy in the present sample were comparable with those of the RCT’s intervention group and consistently lower than in the RCT’s control group. Subgroup analysis comparing rates of patients with a need for QoL therapy in the present sample with both historical controls revealed no significant differences at the primary endpoint at 6 months.

### Patient evaluation

Patients were asked via questionnaire to evaluate the QoL pathway at 6 and 24 months after surgery. The completion rates were 70% (6 months: 39/56) respectively 54% (24 months: 30/56). At 6 months, 74% (29/39) of patients reported that their CP had discussed QoL-profiles with them whereas in 26% (10/39), this happened never or only rarely. At 24 months, the QoL-profile had been discussed with 60% (18/30) of participants compared to 40% (12/30) whose CP had never or only rarely discussed profiles with them. Table 3 shows results of patient evaluations at 6 and 24 months comparing both subgroups.

**Table 3:** Patient evaluations at 6 and 24 months after surgery: subgroup analyzes of participants whose CP “regularly” versus “rarely or never” discussed QoL-profiles with them

	**6 months**			**24 months**		
	QoL-profile regularly discussed with CP (%) (*n* = 29)	QoL-profile rarely/never discussed with CP (%) (*n* = 10)	*P*^a^	QoL-profile regularly discussed with CP (%) (*n* = 18)	QoL-profile rarely/never discussed with CP (%) (*n* = 12)	*p*^**a**^
The discussion of the QoL-profile with the CP was helpful	83	0	** < .001**	78	8	** < .001**
The QoL-profile comprises all dimensions that were relevant for me during the last months	66	70	.56	94	33	**.01**
My other needs (e.g., pain, sorrows, anxiety) were also treated in addition to the diagnosis of breast cancer	72	30	**.04**	94	25	** < .001**
My physicians and therapists found the right treatment strategy for my other needs	69	40	.23	89	17	** < .001**
*Personal benefit by QoL measurements*						
I was regularly asked about my wellbeing	72	50	.25	83	50	.10
My wellbeing and QoL were more often discussed during the medical appointment	55	20	.07	61	8	**.01**
The communication with my CP has improved	24	0	.16	28	8	.36
The relationship with my CP has improved	24	0	.16	33	17	.42
Other benefits	21	0	.31	22	0	.13
No benefit	14	60	**.01**	6	77	** < .001**
The completion of the QoL questionnaire was burdensome	3	10	.45	6	0	1.0

^a^All *p* values derived from *χ*^2^ tests or Fisher’s exacts test if smallest expected cell value was < 5

*p* values < 0.05 are presented in bold face

Six months postoperatively significantly more women whose CP regularly discussed QoL-profiles with them reported that the discussion was helpful (83% vs. 0%, *p* < 0.001) and that their specific needs like pain, sorrows, or anxiety had also been cared for (72% vs. 30%, *p* = 0.04) in comparison to patients whose CP never or only rarely discussed QoL-profiles. At 24 months, regular discussions of QoL-profiles were associated with a higher proportion of women who evaluated these discussions as helpful (78% vs. 8%, *p* < 0.001) and who rated the dimensions of the QoL-profile as personally relevant (94% vs. 33%, *p* = 0.01). They also reported significantly more often that their wellbeing had been discussed more often during the medical appointment (61% vs. 8%, *p* < 001), that their specific needs had been cared for more frequently (94% vs. 25%, *p* < 0.001), and that the right treatment strategy had been found (89% vs. 17%, *p* < 0.001). In contrast, significantly more women whose CP rarely or never discussed QoL-profiles with them reported no benefit by answering QoL questionnaires at 6 (14% vs. 60%, *p* = 0.01) and 24 months (6% vs. 77%, *p* < 0.001).

### Physician evaluation

The physician evaluation at 24 months postoperatively was answered by 52% (17/33) of CPs. Of those, 94% (16/17) found that the QoL pathway was useful for patients with breast cancer with 82% (14/17) reporting that it met the needs of their patients. In detail, 94% (16/17) of CPs discussed the QoL-profile with their patients, 88% (15/17) evaluated it as helpful, and 82% (14/17) reported that the profile had improved patient-physician communication. The dimensions of the QoL-profile were rated as appropriate by all CPs (17/17). Moreover, the address list with QoL therapists was useful for all CPs (17/17) and 94% (16/17) evaluated the EDP-aided paper-based assessment of QoL as feasible. Seventy-six percent (13/17) of CPs wanted to receive the QoL-profile for their other patients with cancer as well.

To assess for bias in the physician survey, we analyzed if CPs whose patients had reported at 6 months in the patient survey that their CP regularly discussed QoL-profiles with them were more likely to participate in the physician survey compared with those CPs who rarely/never discussed QoL-profiles. In total, 26 CPs treated the 39 patients who responded to the patient evaluation at 6 months and 13 of those participated in the physician survey. Participation rates in the physician survey did not differ significantly between CPs who regularly discussed QoL-profiles (9/19, 47%) and CPs who rarely/never discussed QoL-profiles (4/7, 57%; *p* = 1.00).

## Discussion

The refined QoL pathway including the electronic tool *LPro* to present QoL results immediately after measurement was shown to be feasible and useful for inpatient and outpatient care of patients with breast cancer. The vast majority of CPs who participated in the physician evaluation reported that the pathway met patients’ needs and improved patient-physician communication what is in line with previous studies [6–8]. Participation rates of CPs support this finding as only one CP refused participation and another CP dropped out during the study. It was interesting that most CPs preferred to receive the QoL-profile via fax whereas one CP wanted to receive profiles by post and none by e-mail. It has been shown that compliance in practice is higher if fewer new skills and organizational change are needed [24]. Accordingly, new (technical) interventions must be kept as simple as possible for the implementation into the workflow of routine care.

Another important finding is that patients also subjectively benefited from QoL measurements given that their physician discussed QoL results with them. In our study all CPs were recommended to use QoL-profiles for patient-physician communication but a considerable part of patients reported that this happened only rarely or never. A systematic review [25] identified an imbalance in the patient-physician relationship as a potential barrier for the use of patient-reported outcomes that is among others caused by interpersonal characteristics of the doctor like a mere focus on treating a “disease” or dominating decision-making by an authoritarian behavior. It is also possible that CPs considered QoL-profiles during decision-making but did not discuss them directly with their patients. Indeed, discussing QoL results is very important for patients. Thus, we found that participants whose CP regularly discussed QoL-profiles with them evaluated this as helpful and reported that their specific needs were cared for more often. In contrast, patients whose CP never or only rarely discussed QoL-profiles with them significantly more often had no subjective benefit of QoL measurements. This result demonstrates that it is important to integrate QoL diagnosis and therapy into the workflow of aftercare so that QoL can be directly considered and discussed during the medical encounter. QoL measurements at the patient’s home (e.g., via mobile app) that are not discussed with the physician are no adequate alternative. This finding is also important to encourage doctors to use QoL results in patient-physician communication.

Results of the earlier RCT [13] have demonstrated that patients who received QoL diagnosis and therapy showed a faster improvement of their QoL during the first year after surgery. In our sample, rates of patients with a need for QoL therapy were comparable with those of former intervention group patients and consistently lower than in the former control group of the RCT. Differences between groups were not significant because of the small sample size but showed a consistent trend.

In contrast to the earlier RCT [13], patients in the present study did not receive any further reminder if they missed a QoL measurement and there was no regular phone contact between study coordinators and CPs. This resulted in lower follow-up rates in the present sample. However, at the end of the trial at 24 months there were still 71% of participants in the study. Thus, the majority of patients used QoL diagnosis and therapy throughout the first 2 years after surgery. Some patients dropped out during the course of the study because they felt good and had no further need for QoL therapy. The duration of the QoL pathway should be individually adapted based on medical assessment and patients’ preferences.

The study also has some limitations. First, the sample size was relatively small. Initially, a sample size of 200 patients was planned according to the previous RCT with 200 participants [13]. Because of logistical reasons, we decided to include only two regional hospitals that were responsible for patient recruitment in the present study compared with five recruiting hospitals in the RCT so that the sample size of 200 patients could not be reached within the recruitment period. A second limitation was that 36% of patients refused to participate in the study compared with only 18% in the RCT. The specific components of the QoL pathway were identical in both studies (QoL diagnosis and therapy) except of using a tablet computer for the first QoL measurement. This could have reduced the acceptability of the intervention. Participants of the present study were significantly younger compared with RCT patients and with women who refused participation. Wysham et al. [26] found a higher age to be a significant predictor of lower completion rates of electronic patient-reported outcomes in routine cancer care. We tried to address this problem by also offering a paper–pencil version of the questionnaire that was used by 14% of participants. This shows that there needs to be a paper-based alternative to electronic QoL assessments so that more patients can be reached. Furthermore, patient characteristics of historical controls were not completely comparable with those of patients in the present sample (e.g., age, surgical procedure). This may have caused a bias when comparing rates of patients with a need for QoL therapy. In addition, results of the physician survey may be biased by the response rate of 52%. A subgroup analysis showed that participation rates of the physician survey did not differ between CPs who regularly discussed QoL-profiles and those who rarely/never discussed profiles. Thus, highly motivated CPs were not more likely to participate in the survey. Finally, because of multiple testing, results need to be confirmed in future trials.

To our knowledge, this is the first study providing results of QoL measurements immediately to patients and their physicians in a broad inpatient and outpatient setting with a relatively low-tech technical equipment, other than tablet computers or smartphones. In conclusion, patients with breast cancer subjectively benefit from QoL diagnosis and therapy in respect of a higher satisfaction with care given that their doctor discusses QoL results with them. Two previous RCTs [13, 14] have already demonstrated effectiveness of QoL diagnosis and therapy in terms of a better QoL in patients with breast cancer and colorectal cancer. Therefore, QoL diagnosis and therapy should be offered to all cancer patients during treatment and aftercare and regular funding needs to be established, e.g., by health insurances.

## Supplementary Information

Below is the link to the electronic supplementary material.

Supplementary file1 (DOCX 36 KB)

Supplementary file2 (PNG 125 KB)

## Data Availability

The authors have full control of all primary data and agree to allow the journal to review the data if requested.
