# Supplementary material for: Long-term improvement of quality of life in patients with breast cancer: supporting patient-physician communication by an electronic tool for inpatient and outpatient care
Source: Support Care Cancer. 2021 Jun 27;29(12):7865–75. doi: 10.1007/s00520-021-06270-1 (PMC8550515; doi:10.1007/s00520-021-06270-1)
Supplement: Supplementary file 1 — Supplementary file1 (DOCX 36 KB) [file 520_2021_6270_MOESM1_ESM.docx]

**Supplementary Information**

**Supplementary Fig** Automated measurement of QoL via tablet computer and EDP-aided paper-based assessment in inpatient and outpatient care.

**Supplement** Description of server configuration and used software

For making the software ‘*LPro*’ available we set up a dedicated server on which the app can be accessed by using a web browser on a tablet computer. The server is also able to receive the questionnaire by facsimile. Furthermore, we set up a second server that is only available in the local Intranet. The internal server holds the study database containing all personally identifying information, whereas the webserver does not store any personally identifying information for data protection reasons. The questionnaires that are received by the webserver can be transferred to the study database via an XML-Interface and can be linked with the personally identifying information.

We tried to use open source technologies as far as possible. We chose Ubuntu Server as operating system. The software ‘*LPro*’ is using the web development framework ‘Ruby on Rails’ and is deployed with ‘Apache’ and the ‘Phusion Passenger’ module. As a database we use MySQL. The incoming facsimiles are received by the HylaFAX+ fax server and prepared for further processing. The resulting file, which contains the completed questionnaire, is passed to a MATLAB-Script (m-File) and is analyzed using methods of digital image processing. For this the MATLAB ‘Image Processing Toolbox’ is used. The results of the image analysis are stored in MySQL database on the webserver. After that the fully automated evaluation and the creation of the QoL-profile is done with ‘R’. The last step includes an email or facsimile (depending on the user settings) being sent to the authorized clinicians of the hospital and the study coordinators of the Tumor Center Regensburg. The study coordinators also receive log messages for checking the accuracy and quality of data.
