# Supplementary figures and images for: Long-term improvement of quality of life in patients with breast cancer: supporting patient-physician communication by an electronic tool for inpatient and outpatient care
Source: Support Care Cancer. 2021 Jun 27;29(12):7865–75. doi: 10.1007/s00520-021-06270-1 (PMC8550515; doi:10.1007/s00520-021-06270-1)

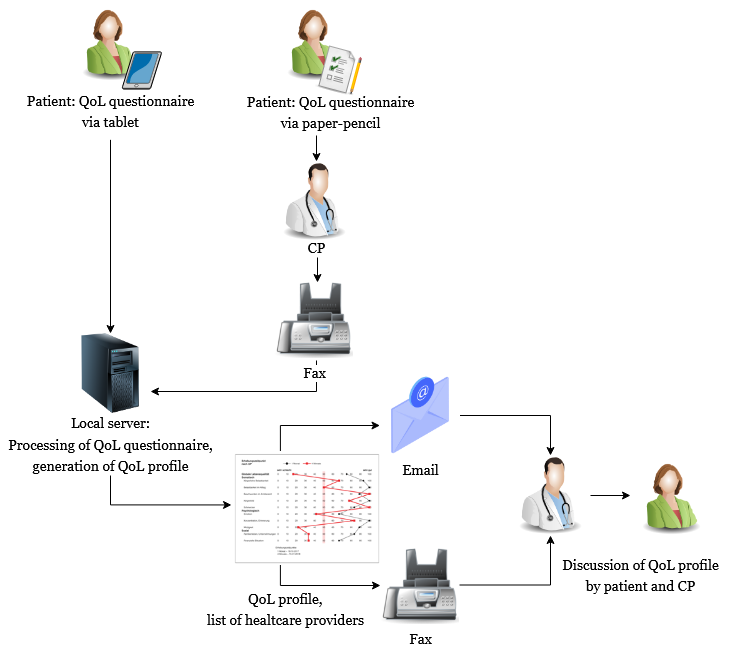

Supplement: Supplementary file 2 — Supplementary file2 (PNG 125 KB) [file 520_2021_6270_MOESM2_ESM.png]
